# Robust Adaptive Beamforming with Sensor Position Errors Using Weighted Subspace Fitting-Based Covariance Matrix Reconstruction

**DOI:** 10.3390/s18051476

**Published:** 2018-05-08

**Authors:** Peng Chen, Yixin Yang, Yong Wang, Yuanliang Ma

**Affiliations:** School of Marine Science and Technology, Northwestern Polytechnical University, Xi’an 710072, China; danielcp9@outlook.com (P.C.); yongwang@nwpu.edu.cn (Y.W.); ylma@nwpu.edu.cn (Y.M.)

**Keywords:** covariance matrix reconstruction, robust adaptive beamforming, sensor position errors, weighted subspace fitting (WSF)

## Abstract

When sensor position errors exist, the performance of recently proposed interference-plus-noise covariance matrix (INCM)-based adaptive beamformers may be severely degraded. In this paper, we propose a weighted subspace fitting-based INCM reconstruction algorithm to overcome sensor displacement for linear arrays. By estimating the rough signal directions, we construct a novel possible mismatched steering vector (SV) set. We analyze the proximity of the signal subspace from the sample covariance matrix (SCM) and the space spanned by the possible mismatched SV set. After solving an iterative optimization problem, we reconstruct the INCM using the estimated sensor position errors. Then we estimate the SV of the desired signal by solving an optimization problem with the reconstructed INCM. The main advantage of the proposed algorithm is its robustness against SV mismatches dominated by unknown sensor position errors. Numerical examples show that even if the position errors are up to half of the assumed sensor spacing, the output signal-to-interference-plus-noise ratio is only reduced by 4 dB. Beam patterns plotted using experiment data show that the interference suppression capability of the proposed beamformer outperforms other tested beamformers.

## 1. Introduction

To extract and enhance the desired signal from a certain direction, beamforming is being widely applied in various fields, including wireless communication, radar, sonar, and audio processing [1,2,3]. As a data-dependent algorithm, adaptive beamforming can adjust the weight vector according to the observation data. The standard Capon beamformer (SCB) [4] is an adaptive beamformer that optimizes performance by minimizing the output power of array while keeping the main beam response distortionless. However, the SCB is sensitive to steering vector (SV) mismatch, especially when the observation data contains the desired signal with a high signal-to-noise ratio (SNR). In practice, SV mismatch usually results from various causes including but not limited to looking direction error, local scattering and sensor displacement, and leads to severe performance degradation of the SCB. Several robust adaptive beamforming algorithms have been developed in the last few decades. The diagonal loading beamformer [5] is a popular modified Capon beamformer. However, its main drawback is its poor performance in a high SNR environment. The uncertainty set-based beamformers in References [6,7,8,9,10] constrain the SV by an ellipsoidal uncertainty set whose optimal weighting vector can be estimated by solving an optimization problem. The two approaches reduce the sensitivity of SV mismatch by manually adding a white noise component to the SCM. The uncertainty set-based beamformers are tradeoff techniques that may degrade beamforming performance under high-SNR conditions.

To remove the desired signal component from observation data, an interference-plus-noise covariance matrix (INCM) reconstruction beamformer was proposed in Reference [11]. In this method, the INCM is reconstructed by integrating the nominal SV and the corresponding Capon power spectrum over the entire angular sector, except the region near the desired signal. Several modified INCM reconstruction-based beamforming algorithms were proposed in References [12,13,14]. However, even when the power spectrum is robustly estimated in the INCM reconstruction procedure, the negative influence of sensor displacement still degrades the performance of reconstruction-based beamformer. Huang et al. recently proposed an annulus uncertainty set based INCM reconstruction beamformer [15] that can provide remarkable performance with an arbitrary array structure. In this approach, the INCM is reconstructed by integrating all possible mismatched SV in the annulus uncertainty set over a small angular sector that only contains interferences, and the reconstructed INCM is robust with random SV errors. Several beamformers proposed in References [16,17] to reconstruct the INCM by removing the estimated desired signal components from the SCM directly. Although the beamformers in References [15,16,17] can overcome slight random SV mismatch, in some cases the main challenge is a severe sensor position error. For example, in the experiments of ocean acoustics, sensors are usually manually attached on a long flexible cable at a large sensor spacing, and the tough marine environment causes the cable to stretch and deform unevenly, which triggers severe sensor displacement. in this case, the abovementioned INCM-based beamformers may suffer from severe performance degradation.

In this paper, we propose a robust adaptive beamformer called the weighted subspace fitting-based INCM reconstruction and SV estimation beamformer (WSF–REB) for linear arrays. The sensor position errors and corrected interference SVs are estimated by weighing the proximity between the space spanned by the possible mismatched SV of the desired signal and interference with signal subspace. The INCM can be reconstructed using estimated sensor position errors and the corrected SVs in the interferences angular sector. Compared with the other robust INCM-based methods, the proposed WSF-REB specifically constructs a mismatched SV set for linear arrays with sensor displacement, and the WSF-REB estimates the sensor position error by solving an iterative univariate optimization problem. Numerical examples and experiment result validate the robust performance of the WSF–REB with imprecise array geometry, a novel algorithm that can estimate in a satisfactory manner.

## 2. Signal Model and Background

Assume that an array with *M* omnidirectional sensors receives narrowband signals from several far-field sources, in which the observation data at *k*th snapshots is given by:

(1) $$x(k)=a_{0}s_{0}(k)+\sum_{q=1}^{Q}a_{q}s_{q}(k)a_{q}+n(k)$$

where $s_{0}(k)$, $s_{q}(k)$, and n(k) denote the waveform of the desired signal, the *q*th interference, and the additive white Gaussian noise component, respectively. *Q* is the number of interferences, while $a_{0}$ and $a_{q}$ are the SVs of the desired signal and interference, respectively. We assume the desired signal, interferences, and noise to be uncorrelated.

Therefore, the beamformer output can be written as:

(2) $$y(k)=w^{H}x(k)$$

where w is the N×1 complex weight vector and ${\left(\cdot \right)}^{H}$ denotes the Hermitian transpose operator.

To analyze the performance of adaptive beamformers quantitatively, the output signal-to-interference-plus-noise ratio (SINR) is defined as:

(3) $$SINR=\frac{\sigma_{s}^{2}|w^{H}a_{0}{|}^{2}}{w^{H}R_{i+n}w}$$

where $\sigma_{s}=E\{|s_{0}{|}^{2}\}$ is the power of the desired signal, E{·} denotes the statistical expectation operator, and $R_{i+n}=E\{(x_{\mathrm{int}}(k)+n(k)){\left(x_{\mathrm{int}}(k)+n(k)\right)}^{H}\}$ is the INCM.

The maximization of Equation (3) can be equivalent to the minimization of output power under the condition that the response of the main beam remains distortionless, which can be formed as:

(4) $$\underset{w}{\min}\;w^{H}R_{i+n}w,\;s.t.\;w^{H}a_{0}=1$$

By applying the Lagrangian multiplier to Equation (4), the Capon beamformer weight vector $w_{\mathrm{Capon}}$ can be obtained as:

(5) $$w_{\mathrm{Capon}}=\frac{R_{i+n}^{-1}a_{0}}{a_{0}^{H}R_{i+n}^{-1}a_{0}}$$

Signal-free observation data are usually unavailable in real applications, thus $R_{i+n}^{-1}$ can be replaced by the SCM $\hat{R}=(1/K)\sum_{k=1}^{K}x(k)x^{H}(k)$. Therefore, the SCM inversion (SMI) beamformer weight vector can be obtained as $w_{\mathrm{SMI}}={\hat{R}}^{-1}a_{0}/a_{0}^{H}{\hat{R}}^{-1}a_{0}$. To increase the robustness of the SMI beamformer, the diagonal loading sample covariance matrix inversion (LSMI) technique [5] was proposed as:

(6) $$w_{\mathrm{LSMI}}=\frac{{\left(\hat{R}+\gamma I\right)}^{-1}a_{0}}{a_{0}^{H}{\left(\hat{R}+\gamma I\right)}^{-1}a_{0}}$$

where γ is the diagonal loading level. As γ increases, the LSMI becomes increasingly robust at the cost of decreasing beamformer performance. Moreover, the optimal diagonal loading level, which balances the robustness and high performance, cannot be easily chosen.

Several robust beamformers based on SV uncertainty have been proposed in recent decades. One of the most popular algorithms is the robust Capon beamformer (RCB) [7], which can be formed as:

(7) $$\underset{a}{\min}\;a^{H}R^{-1}a,\;s.t.\;\left\|a-{\bar{a}}_{0}\right\|\leq \varepsilon$$

where ${\bar{a}}_{0}$ is the nominal SV of the desired signal, and ε is the radius of the uncertainty set.

The abovementioned beamformers are sensitive to SV mismatch due to signal direction error, sensor displacement and other causes when the desired signal exists in the observation data. To form a *quasi*-signal-free condition, [11] proposed an INCM reconstruction method that enhances beamformer performance by removing the desired signal component from the observation data. Assuming that Θ is an angular sector that contains the desired signal, the INCM can be reconstructed as:

(8) $${\tilde{R}}_{i+n}=\int_{\Theta_{\mathrm{int}}}\hat{P}(\theta )\bar{a}(\theta ){\bar{a}}^{H}(\theta )d\theta$$

where $\bar{\Theta }$ denotes the complement sector of Θ. Here, all interferences are in $\bar{\Theta }$, and $\hat{P}(\theta )$ is the estimated Capon power spectrum at direction θ. To address the sparsity of interferences in the whole spatial domain, a sparse interference covariance matrix reconstruction method was proposed in Reference [12] as:

(9) $${\tilde{R}}_{i+n}=\int_{\Theta_{\mathrm{int}}}\hat{P}(\theta )\bar{a}(\theta ){\bar{a}}^{H}(\theta )d\theta +\sigma_{n}^{2}I$$

where $\Theta_{\mathrm{int}}$ denotes the angular sector that contains only the nearby angular grids of interferences, and $\sigma_{n}^{2}$ is the noise power that can be estimated as the minimum eigenvalue of $\hat{R}$.

An INCM-based beamformer with an annulus uncertainty set [15] was proposed to overcome random SV mismatch, and the INCM can be formed as:

(10) $${\tilde{R}}_{i+n}\approx \frac{1}{2}\sum_{i}^{I}\sum_{l=1}^{L}\frac{{\tilde{a}}_{il}{\tilde{a}}_{il}^{H}}{{\tilde{a}}_{il}^{H}{\hat{R}}^{-1}{\tilde{a}}_{il}}+\sigma_{n}^{2}I$$

where ${\tilde{a}}_{il}=\bar{a}(\theta_{i})+e_{l}$ denotes the *l*th possible SV at the *i*th angular grid in $\Theta_{\mathrm{int}}$, *I* is the number of angular grids in $\Theta_{\mathrm{int}}$, $e_{l}$ is the *l*th possible SV error, and *L* denotes the number of possible SV for one angular grid.

The INCM-based beamformers are robust in certain imperfect environments, such as those with signal direction error, local scattering, and multipath effect. However, the reconstruction of covariance matrix is sensitive to interference SV mismatch, especially when the mismatch is caused by imprecise array geometry. Although the possible SV set is constructed to combat random SV errors in Reference [15], the remission seems mild, and such INCM-based beamformers may perform worse than SCM-based techniques when the SV mismatch is dominated by severe sensor position errors.

## 3. Proposed WSF-REB

In this section, we propose a WSF-based covariance matrix reconstruction method for linear arrays to overcome sensor displacement. With sensor position errors, the INCM is more sensitive to the component $\bar{a}(\theta ){\bar{a}}^{H}(\theta )$ than $\hat{P}(\theta )$ in Equation (9). Instead of increasing the accuracy of power spectrum or utilizing Capon power spectrum to weigh the constructed possible SV set, we construct a new form of possible mismatched SV. Then, we transform WSF from a direction of arrival (DOA) estimation method into a method that estimates sensor position errors with the possible mismatched SV set. Using the obtained sensor position errors, we reconstruct the INCM and renew the SV of desired signal.

The SCM can be eigen-decomposed as:

(11) $$\hat{R}=\sum_{i=1}^{M}\lambda_{i}e_{i}e_{i}^{H}=V_{s}D_{s}V_{s}^{H}+V_{n}D_{n}V_{n}^{H}$$

where $\lambda_{i},i=1,\cdots ,M$ represents the eigenvalue of $\hat{R}$ in descending order, while $e_{i}$ denotes the corresponding eigenvector. The diagonal matrix $D_{s}=\mathrm{diag}[\lambda_{1},\cdots ,\lambda_{P}]$ contains the *P*-dominant eigenvalues of $\hat{R}$, and $V_{s}$ consists of corresponding eigenvectors.

When the snapshot number of observation data is infinite, the subspace spanned by the actual SVs of the desired signal and interferences equals to the signal subspace, which can be expressed as:

(12) $$\mathrm{span}\{V_{s}\}=\mathrm{span}\{a(\theta_{0}),a(\theta_{1}),\cdots ,a(\theta_{Q})\}=\mathrm{span}\{A\}$$

where $\theta_{q},\;q=0,1,\cdots ,Q$ are the DOAs of the desired signal and interferences. However, $\mathrm{span}\left\{V_{s}\right\}$ will no longer equal span{A} when the snapshot number is finite, and the WSF method is introduced to quantitatively analyze the proximity of $\mathrm{span}\left\{V_{s}\right\}$ and span{A}. When SV mismatch exists, the proximity of two space decreases. Therefore, the estimated SV set of the desired signal and interferences can be formed as:

(13) $$A(\hat{\theta })=[\begin{array}{cccc} \bar{a}({\hat{\theta }}_{0}) & \bar{a}({\hat{\theta }}_{1}) & \cdots & \bar{a}({\hat{\theta }}_{Q}) \end{array}]$$

where ${\hat{\theta }}_{q},\;q=0,1,\cdots ,Q$ are the roughly estimated DOAs of the desired signal and interferences in Θ and $\bar{\Theta }$. Due to the existence of SV error, the estimated DOAs may not be accurate. Nevertheless, the error of $A(\hat{\theta })$ is still dominated by severe sensor position errors.

For a linear array, we assume that the sensor displacement is one-dimensional. In other words, the actual positions of sensors are still on the straight line of the assumed linear array. To estimate the sensor position errors in a linear array, we define the error vector of SV at direction ${\hat{\theta }}_{q}$ as:

(14) $$a_{e}(d_{e},{\hat{\theta }}_{q})=e^{jk_{w}d_{e}\sin{\hat{\theta }}_{q}}$$

where $j=\sqrt{-1}$ denotes the imaginary unit, $k_{w}$ is the wavenumber, $d_{e}={\left[d_{1},d_{2},\cdots ,d_{M}\right]}^{T}\in {\mathbb{R}}^{M\times 1}$ is the possible sensor position errors vector, ${\left(\cdot \right)}^{T}$ denotes the transpose operator, and ℝ represents the real number field. Then, the possible mismatched SV we proposed can be constructed as:

(15) $$a(d_{e},{\hat{\theta }}_{q})=a_{e}(d_{e},{\hat{\theta }}_{q})⊙\bar{a}({\hat{\theta }}_{q})$$

where ⊙ denotes the dot product. The SV is constructed by referencing the first sensor of the array; thus, the position error of the first sensor is 0. Here, the possible mismatched SV in Equation (15) can be rewritten as:

(16) $$a(d_{e},{\hat{\theta }}_{q})=e^{jk_{w}{\left[0,d_{2},\cdots ,d_{M}\right]}^{T}\sin{\hat{\theta }}_{q}}⊙\bar{a}({\hat{\theta }}_{q})$$

Here, the SV set of the desired signal and interferences with possible sensor position errors can be updated as:

(17) $$A(d_{e},\hat{\theta })=[a(d_{e},{\hat{\theta }}_{0}),a(d_{e},{\hat{\theta }}_{1}),\cdots ,a(d_{e},{\hat{\theta }}_{Q})]$$

The WSF method is sensitive to SV, it was originally applied to DOA estimation [18,19] when the SV is a univariate function of angle. The SV error in Equation (14) can be seen as a dualistic function of signal incident angles and sensor displacement. The WSF method can quantitatively analyze the proximity of $V_{s}$ and the space spanned by the SV set with sensor position errors in Equation (17). Once the DOAs of desired signal and interferences are roughly estimated and fixated, the SV errors in Equation (14) can be expressed as univariate function of sensor position errors, and the sensor position errors can be estimated by exploiting the sensitivity of WSF. Therefore, the estimated sensor position errors can be obtained by solving an optimization problem as:

(18) $${\hat{d}}_{e}=\underset{d_{e}}{\min}{\left\|V_{s}W^{1/2}-A(d_{e},\hat{\theta })T\right\|}_{F}$$

where ${\left\|\cdot \right\|}_{F}$ denotes the Frobenius norm, and under the condition of lowest asymptotic variance, the positive definite weighting matrix W is formed as:

(19) $$W={\left(D_{s}-\sigma_{n}^{2}I\right)}^{2}D_{s}^{-1}$$

We solve Equation (18) for T by fixating $A(d_{e},\hat{\theta })$, then we substitute T back into Equation (18), thereby obtaining:

(20) $${\hat{d}}_{e}=\underset{d_{e}}{\min}\;\mathrm{tr}\{P^{⊥}V_{s}WV_{s}^{H}\}$$

where $P^{⊥}=I-A(d_{e},\hat{\theta }){\left[A^{H}(d_{e},\hat{\theta })A(d_{e},\hat{\theta })\right]}^{-1}A^{H}(d_{e},\hat{\theta })$, and tr{·} denotes the matrix trace. Directly solving Equation (20) is difficult because it is a multivariable problem. However, because of the independence of sensor position errors, it is reasonable to estimate one variable while other variables remain fixated. Therefore, the multivariable optimization problem can be rewritten as:

(21) $${\hat{d}}_{m}=\underset{d_{m}}{\min}\mathrm{tr}\{P_{m}^{⊥}V_{s}WV_{s}^{H}\}$$

where ${\hat{d}}_{m}$ denotes the *m*th estimated sensor position error, $P_{m}^{⊥}=I-{\hat{A}}_{m}{\left({\hat{A}}_{m}^{H}{\hat{A}}_{m}\right)}^{-1}{\hat{A}}_{m}^{H}$, ${\hat{A}}_{m}=[{\hat{a}}_{m}({\hat{\theta }}_{0}),{\hat{a}}_{m}({\hat{\theta }}_{1}),\cdots ,{\hat{a}}_{m}({\hat{\theta }}_{Q})]$ is the *m*th SV set with sensor position errors, and the possible mismatched SV with the position error of the *m*th sensor is:

(22) $${\hat{a}}_{m}({\hat{\theta }}_{q})=e^{jk_{w}{\left[0,\cdots ,{\hat{d}}_{m-1},d_{m},0_{1\times (M-m)}\right]}^{T}\sin{\hat{\theta }}_{q}}⊙\bar{a}({\hat{\theta }}_{q})$$

where $0_{1\times (M-m)}$ denotes an 1×(M−m) zero matrix. Then the sensor position errors can be computed as summarized in Table 1. The optimization problem in Equation (21) can be efficiently solved by optimization methods such as Newton down-hill method and gradient descent method, etc.

With the well-estimated sensor position errors, the INCM can be reconstructed as:

(23) $${\hat{R}}_{i+n}=\int_{\Theta_{\mathrm{int}}}\frac{E(\theta )⊙(\bar{a}(\theta ){\bar{a}}^{H}(\theta ))}{{\bar{a}}^{H}(\theta )(E(\theta )⊙{\hat{R}}^{-1})\bar{a}(\theta )}d\theta +\sigma_{n}^{2}I$$

where $E(\theta )=a_{e}({\hat{d}}_{e},\theta )a_{e}^{H}({\hat{d}}_{e},\theta )$ is the error matrix at direction θ, and $a_{e}({\hat{d}}_{e},\theta )=e^{jk_{w}{\hat{d}}_{e}\sin\theta }$ denotes the estimated error vector of SV at direction θ. With the reconstructed INCM ${\hat{R}}_{i+n}^{-1}$ from Equation (23), we can correct the SV of desired signal by using the similar idea of that presented in Reference [11], which maximize the beamformer output power by solving a optimization problem.

(24) $$\begin{array}{l} \underset{e_{⊥}}{\min}\;{\left({\bar{a}}_{0}+e_{⊥}\right)}^{H}{\hat{R}}_{i+n}^{-1}({\bar{a}}_{0}+e_{⊥})\\ s.t.\;{\bar{a}}_{0}^{H}e_{⊥}=0\;,\;{\left({\bar{a}}_{0}+e_{⊥}\right)}^{H}{\hat{R}}_{i+n}({\bar{a}}_{0}+e_{⊥})\leq \;{\bar{a}}_{0}^{H}{\hat{R}}_{i+n}{\bar{a}}_{0} \end{array}$$

where $e_{⊥}$ is the orthogonal component of the mismatch vector which is kept by the equality constraint, the inequality constraint is used for preventing the corrected SV of desired signal ${\hat{a}}_{0}={\bar{a}}_{0}+e_{⊥}$ from converging to interferences. This problem is obviously convex and can be easily solved by various optimization toolboxes. Therefore, the proposed WSF–REB can be designed as:

(25) $$w_{\text{WSF-REB}}=\frac{{\hat{R}}_{i+n}^{-1}{\hat{a}}_{0}}{{\hat{a}}_{0}^{H}{\hat{R}}_{i+n}^{-1}{\hat{a}}_{0}}$$

The procedure of the proposed WSF-REB algorithm is summarized in Table 2.

In the INCM reconstruction procedure, where the complexity is dominated by sensor position errors estimation, the complexity can be expressed as $Ο(\mu M^{2}Q^{2})$, where μ is the number of optimization iterations in Equation (21). In general, Q≤M when the number of signals is smaller than the number of sensors, and μ depends on the optimization method adopted and the condition of convergence. The SV estimation in Equation (24) can be efficiently solved at a complexity cost of $Ο(M^{3.5})$. Hence the complexity of our algorithm is $Ο(\max(\mu M^{2}Q^{2},M^{3.5}))$.

## 4. Numerical Examples

In the following numerical examples, a 10-sensor uniform linear array spaced half-wavelength is considered, and we assume that the sensors are calibrated, which are free of gain-phase errors. Two interferences, both with interference-to-noise ratios (INR) fixed at 20 dB, impinge from $\theta_{1}=-25^{\circ }$ and $\theta_{2}=35^{\circ }$. The desired signal impinges from $\theta_{0}=15^{\circ }$. The desired signal and interferences are generated from zero means complex Gaussian noises and therefore spatially and temporally independent. In the case of SINR versus SNR, the number of snapshots is fixed at *K* = 30, and in the case of SINR versus snapshots number, the SNR is fixed at 10 dB.

The proposed beamformer is compared with five beamformers, namely the INCM reconstruction estimation beamformer (REB) [11], the annulus uncertainty set-based INCM reconstruction estimation beamformer (Annulus–REB) [15], the IAA-based INCM reconstruction beamformer (IAA–RB) [14], the LSMI beamformer [5], and RCB [7]. The optimal SINR is calculated as ${SINR}_{\mathrm{opt}}=\sigma_{s}^{2}a_{0}^{H}R_{i+n}^{-1}a_{0}$.

We assume that the angular sector for REB, IAA–RB and the proposed beamformer is $\Theta =[10^{\circ },20^{\circ }]$. For Annulus–REB, the parameter is $\varepsilon =\sqrt{0.1}$ and the interference angular sector is $\Theta_{\mathrm{int}}=[-30^{\circ },-20^{\circ }]\cup [30^{\circ },40^{\circ }]$. The diagonal level for LSMI is 10, the constraint parameter for RCB is ε=0.3M, and the interference angular sector for the proposed beamformer is the same as that for Annulus–REB. For the proposed WSF–REB, the estimated DOAs ${\hat{\theta }}_{q}$ are obtained from the Capon power spectrum identified by a peak picking algorithm. The results of all numerical examples are obtained from an average of 200 Monte Carlo simulations.

### 4.1. Example 1: Exactly Known DOAs of the Desired Signal and Interference

**Figure 1:**
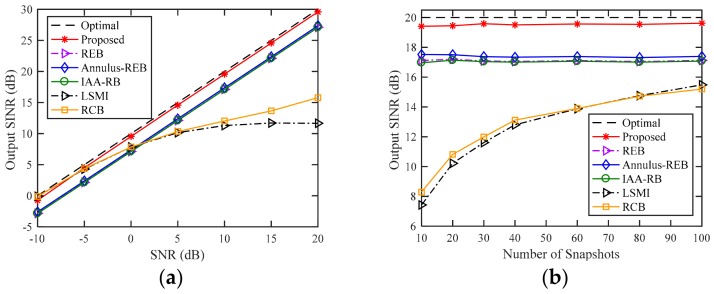
Output SINR versus (**a**) SNR (**b**) number of snapshots with exactly known DOAs and random sensor displacement.

In the first example, we consider an ideal case that the DOAs of all signals are precisely known. Hence, we can skip step 1 in Table 2, and estimate the sensor position errors directly. It can be noted that even with accurate DOAs, the desired signal component in the training still degrades the SCM-based beamformers, and the other tested INCM-based beamformers still suffer from SV errors result from sensor displacement. The position errors of the sensors (except the first one) are normally distributed in $N(0,{\left(0.02\lambda \right)}^{2})$, where λ is the wavelength. It can be seen from Figure 1a that although the proposed beamformer is an INCM-based beamformer, the performance of the proposed beamformer is almost always equal to the optimal SINR for all values of SNR. REB, Annulus-REB, and IAA-RB present nearly 3 dB degradation in the output SINR for all values of SNR compared to the optimal SINR. When the SNR is lower than 0 dB, these three beamformers perform even worse than the SCM-based beamformer. However, the performance of LSMI and RCB degrade with the increase of SNR, when SNR = 10 dB, the SCB-based beamformer suffer from almost 8 dB SINR degradation compared to the optimal SINR. In Figure 1b, the output SINR versus the number of snapshots when SNR is fixed at 10 dB is demonstrated. The output SINR of LSMI and RCB presents nearly 12 dB degradation for 10 snapshots compared to the optimal SINR, and the performance improves with the increase in the number of snapshots. The performance of the INCM-based beamformers is robust with the different number of snapshots, and the proposed beamformer presents nearly 2.5 dB improvement compared to REB, Annulus-REB, and IAA-RB for all snapshots number.

### 4.2. Example 2: Additional SV Mismatch due to Random Signal Look Direction Error

**Figure 2:**
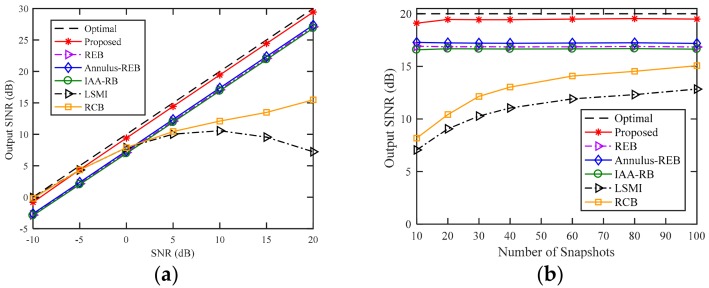
Output SINR versus (**a**) SNR (**b**) number of snapshots with random signal direction error and sensor displacement.

In the second example, we consider the effect of random signal direction error on the output SINR in the case of sensor displacement. The setting of sensor displacement is the same as the one in Example 1. We assume that the random direction errors of the desired signal and interferences are subject to uniform distribution in $\left[-2^{\circ },2^{\circ }\right]$ for each simulation. In other words, the directions of the signals change from one run to another but remain fixed for all snapshots. Compared with Figure 1, the performances of RCB, REB, Annulus-REB and IAA-RB in Figure 2 are nearly unchanged, and the output SINR of LSMI degrades when SNR is larger than 10 dB. The performance of the proposed beamformer in Figure 2 suffer from a slight degradation compared to Figure 1, this is because the random direction error results in inaccurate DOA estimation of desired signal and interferences, and then causes inaccurate sensor position error estimation. However, the influence of signal direction errors on the performance of WSF-REB is limited, the proposed beamformer can still provide a robust and satisfactory performance compared to other tested beamformers.

### 4.3. Example 3: Additional SV Mismatch due to Incoherent Local Scattering

**Figure 3:**
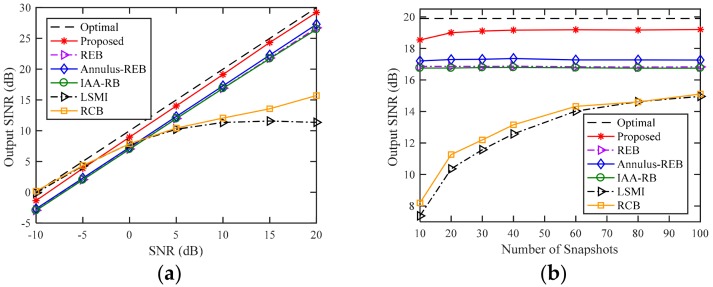
Output SINR versus (**a**) SNR (**b**) number of snapshots with incoherent local scattering and random sensor displacement.

In this example, we consider the influence of SV mismatch caused by local scattering on array output SINR in the case of sensor displacement. According to Reference [20], the desired signal has a time-varying SV. Therefore, the received desired signal can be modeled as:

(26) $${\tilde{s}}_{0}(k)=a_{0}s_{0}(k)+\sum_{p=1}^{4}a(\theta_{p})s_{p}(k)$$

where $a_{0}$ is the SV of the direct path from $\theta_{0}=15^{\circ }$, $a(\theta_{p})$ denotes the SV of the locally scattered signals from $\theta_{p}$ and is uniformly distributed in $\left[\theta_{0}-2^{\circ },\theta_{0}+2^{\circ }\right]$, $s_{0}(k)$ and $s_{p}(k),\;p=1,2,3,4$ are generated from additive complex Gaussian noise, and the sensor displacement settings are the same as those in Example 1. In Figure 3, the performances of all tested beamformers except for the WSF-REB are almost the same as in Figure 1. However, the local scattering of desired signal causes that the signal subspace contains includes not only desired signal and interferences but also local scattered signals. The scattered signals in the signal subspace result in inaccurate sensor position error estimations during the subspace fitting procedure, and then make the INCM inaccurately reconstructed. In Figure 3, the proposed beamformer suffers from nearly 1 dB degradation in the output SINR for all values of SNR and number of snapshots compared to the performance of WSF-REB in Figure 1. However, the proposed beamformers still outperform other tested INCM-based beamformers with an advantage of nearly 2 dB for all value of SNR and snapshots number in the case of additional incoherent local scattering.

### 4.4. Influence of Sensor Displacement

**Figure 4:**
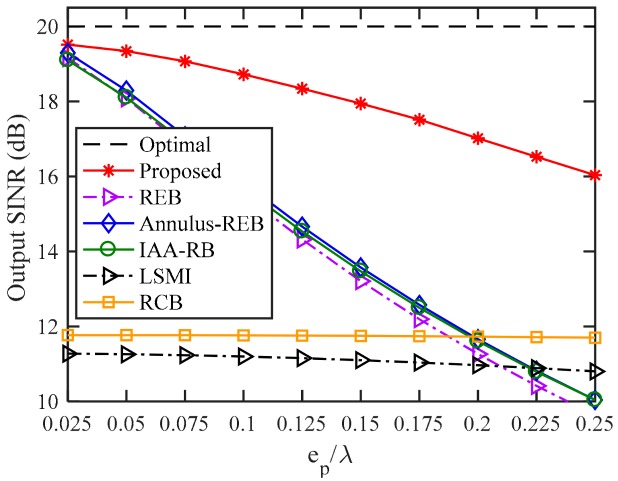
Output SINR versus upper boundary of sensor position error.

In this sub-section, we investigate the influence of sensor displacement on the performance of the proposed beamformer. The position errors of the sensors (except the first one) are uniformly distributed in the interval $\left(-e_{p},e_{p}\right)$, where $e_{p}$ is the upper boundary. The SNR is fixed at 10 dB, and Figure 4 shows that with the increase of sensor position errors, the output SINR of RCB and LSMI stay at nearly 12 dB and 11 dB, respectively. The performances of all INCM-based beamformers deteriorate as sensor position errors increase. When the sensor position error boundary is larger than 0.2λ, REB, Annulus–REB, and IAA–RB perform worse than the SCM-based adaptive beamformers. However, with estimated sensor position errors, the proposed WSF–REB mitigate the performance degradation caused by sensor displacement, even when the upper boundary is up to 0.25λ (half of assumed sensor spacing), the proposed beamformer can still provide an output SINR of 16 dB, while the output SINRs of REB, Annulus-REB and IAA-RB are only nearly 10 dB.

## 5. Experiment Results

**Figure 5:**
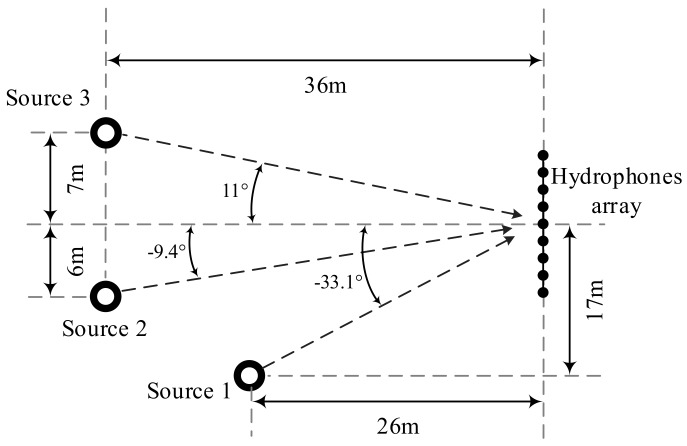
Output SINR versus upper boundary of sensor position error.

In this section, an experiment result is demonstrated to verify the performance of proposed algorithm. The experiment was taken place in a deep lake, the position settings of sources and hydrophone array are shown in Figure 5, and the azimuth of sources can be easily calculated.

The desired signal is a series of CW pulses at 2500 Hz that is generated from source 1, and it impinges from $\theta_{0}=-33.1^{\circ }$. The interferences from source 2 and source 3 are generated from band-pass (2490~2510 Hz) filtered white Gaussian noises, and impinge from $\theta_{1}=-9.4^{\circ }$ and $\theta_{2}=11^{\circ }$, respectively. A horizontal linear array with 9 hydrophones are used to receive the signals from all sources. The hydrophones are installed on an inflexible linear trestle at a precise spacing of d=0.3 m, and all the sensors are pre-calibrated and free of gain-phase error. The sample frequency is $f_{s}=20,800\;\mathrm{Hz}$, the number of snapshots are *K* = 20,000, and the signal propagation speed is c≈1510 m/s. The constraint angular sector is $\Theta =[-35^{\circ },-33^{\circ }]$, the interference angular sector is $\Theta_{\mathrm{int}}=[-11^{\circ },-7^{\circ }]\cup [9^{\circ },13^{\circ }]$.

**Figure 6:**
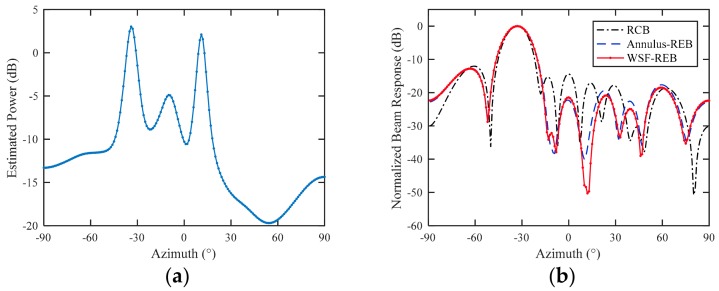
Experiment results. (**a**) Estimated Capon spectrum using assumed nominal SVs; (**b**) beam patterns of tested beamformers in the case of sensor position error.

To explore the performance of the tested beamformers in the case of sensor displacement, the nominal SVs used to calculate the weight vector w are calculated by using assumed sensor positions, which are obtained by adding slight position errors on the actual sensor positions. In Figure 6a, we estimate the Capon power spectrum for the proposed WSF–REB, the estimated DOAs of desired signal and interferences are ${\hat{\theta }}_{0}=-33.8^{\circ }$, ${\hat{\theta }}_{1}=-9.8^{\circ }$, ${\hat{\theta }}_{2}=11.3^{\circ }$, and the position errors in Figure 6 are listed in Table 3.

To compare the performances of proposed beamformer with the other tested beamformers, the beam responses of beamformers are calculated using actual SV as:

(27) $$B(\theta )={\left\|w^{H}a(\theta )\right\|}^{2}$$

According to the simulation examples, the RCB performs better than the LSMI, and the Annulus-REB is better than the REB and IAA-RB. Therefore, we choose RCB as a representative SCM-based beamformer and Annulus-REB as a representative INCM-based beamformer for comparison with the proposed beamformer. Figure 6b shows the beam patterns of RCB, Annulus-REB and the proposed WSF-REB for a typical setting of position errors. When sensor position errors exist, RCB successfully keeps the main lobe towards the direction of desired signal, but it fails to suppress the interferences. The proposed WSF-REB precisely suppress two interferences, and it performs obviously better than the Annulus-REB. It is worth noting that different position errors settings will result in different beam patterns. Although the WSF-REB has almost the same beam pattern as Annulus-REB in the worst case scenario, the interference suppression capability of WSF-REB is much better than the Annulus-REB in most of the outcomes.

## 6. Conclusions

In this paper, we devise a robust algorithm that uses WSF-based INCM reconstruction to address adaptive beamforming with imprecise array geometry of linear arrays. By fitting the space spanned by the possible mismatched SVs of the desired signal and interferences with the signal subspace from SCM, the accurate position error of each sensor can be estimated by solving an iterative optimization problem. After reconstructing the INCM using the estimated sensor position errors, the SV of the desired signal can be estimated by maximizing the beamformer output power. Numerical examples and experiment results illustrate the robust performance of the proposed beamformer.

## Figures and Tables

**Table 1:** Sensor position errors estimation algorithm.

$\begin{array}{l} \mathrm{for}\;m=2:M\\ \;\mathrm{for}\;q=0:Q\\ \;\;{\hat{a}}_{m}({\hat{\theta }}_{q})=\exp\{jk_{w}{\left[0,\cdots ,{\hat{d}}_{m-1},d_{m},0_{1\times (M-m)}\right]}^{T}\sin{\hat{\theta }}_{q}\}⊙\bar{a}({\hat{\theta }}_{q})\\ \;\mathrm{end}\\ \;{\hat{A}}_{m}=[{\hat{a}}_{m}({\hat{\theta }}_{0}),{\hat{a}}_{m}({\hat{\theta }}_{1}),\cdots ,{\hat{a}}_{m}({\hat{\theta }}_{Q})]\\ \;P_{m}^{⊥}=I-{\hat{A}}_{m}{\left({\hat{A}}_{m}^{H}{\hat{A}}_{m}\right)}^{-1}{\hat{A}}_{m}^{H}\\ \;{\hat{d}}_{m}=\min\;\mathrm{tr}\{P_{m}^{⊥}V_{s}WV_{s}^{H}\}\\ \mathrm{end} \end{array}$

**Table 2:** Proposed WSF-REB algorithm.

**Step 1:** Obtain the estimated DOAs ${\hat{\theta }}_{q}$ within the angular sector Θ and $\bar{\Theta }$.
**Step 2:** Estimate the sensor position error ${\hat{d}}_{e}$ in Table 1.
**Step 3:** Reconstruct the INCM in Equation (23) using the estimated sensor position error.
**Step 4:** Estimate the SV of desired signal ${\hat{a}}_{0}={\bar{a}}_{0}+e_{⊥}$ using the estimated ${\hat{R}}_{i+n}$.
**Step 5:** Calculate the weight vector $w_{\text{WSF-REB}}$ using Equation (25).

**Table 3:** Sensor position errors for Figure 6.

Senor Number	1	2	3	4	5	6	7	8	9
Position error	0	−0.0301	0.0087	0.0162	0.0116	0.0153	−0.0236	0.0117	−0.0116
